# Virological failure of staggered and simultaneous treatment interruption in HIV patients who began Efavirenz-based regimens after allergic reactions to nevirapine

**DOI:** 10.1186/1742-6405-10-4

**Published:** 2013-01-25

**Authors:** Krittaecho Siripassorn, Weerawat Manosuthi, Aranya Pakdee, Sunanta Natprom, Anuttra Chaovavanich, Narongsak Hengphadpanadamrong, Khobchok Woratanarat, Aroon Lueangniyomkul, and Kiat Ruxrungtham

**Affiliations:** 1 Department of Internal Medicine, Bamrasnaradura Infectious Diseases Institute, 126 Tiwanon Road, Nonthaburi, 11000, Thailand; 2 Department of Medicine, Faculty of Medicine, Division of Allergy and Clinical Immunology, Chulalongkorn University, Bangkok, Thailand

**Keywords:** Nevirapine hypersensitivity or allergy, Efavirenz, Simultaneous interruption, Staggered interruption, Thai

## Abstract

**Objective:**

The objective of this work was to study the virological outcomes associated with two different types of treatment interruption strategies in patients with allergic reactions to nevirapine (NVP). We compared the virological outcomes of (1) HIV-1-infected patients who discontinued an initial NVP-based regimen because of cutaneous allergic reactions to NVP; different types of interruption strategies were used, and second-line regimen was based on efavirenz (EFV); and (2) HIV-1-infected patients who began an EFV-based regimen as a first-line therapy (controls).

**Methods:**

This retrospective cohort included patients who began an EFV-based regimen, between January 2002 and December 2008, as either an initial regimen or as a subsequent regimen after resolving a cutaneous allergic reaction against an initial NVP-based regimen. The study ended in March 2010. The primary outcome was virological failure, which was defined as either (a) two consecutive plasma HIV-1 RNA levels >400 copies/mL or (b) a plasma HIV-1 RNA level >1,000 copies/mL plus any genotypic resistance mutation.

**Results:**

A total of 559 patients were stratified into three groups: (a) Simultaneous Interruption, in which the subjects simultaneously discontinued all the drugs in an NVP-based regimen following an allergic reaction (n=161); (b) Staggered Interruption, in which the subjects discontinued NVP treatment while continuing nucleoside reverse transcriptase inhibitor (NRTI) backbone therapy for a median of 7 days (n=82); and (c) Control, in which the subjects were naïve to antiretroviral therapy (n=316). The overall median follow-up time was 43 months. Incidence of virological failure in Simultaneous Interruption was 12.9 cases per 1,000 person-years, which trended toward being higher than the incidences in Staggered Interruption (5.4) and Control (6.6). However, differences were not statistically significant.

**Conclusions:**

Among the patients who had an acute allergic reaction to first-line NVP-based therapy and later began an EFV-based regimen, virological outcomes resulting from a staggered interruption of treatment (with a continuation of NRTI backbone therapy for 7 days after discontinuing NVP) did not differ from those of the patients who began an EFV-based regimen as their initial therapy (Control). However, the virological failure of Simultaneous Interruption was possibly higher than those of Control and Staggered Interruption.

## Introduction

Non-nucleoside analog reverse transcriptase inhibitor (NNRTI)-based regimens are currently recommended as a first-line therapy for HIV-1-infected patients because of their virological potency, lower risk of drug interactions and lower cost, as well as the availability of fixed-dose combinations. Nevirapine (NVP) is a first-line NNRTI recommended for antiretroviral-naïve patients, according to the European AIDS Clinical Society (EACS) guidelines [1]. NVP is also an acceptable first-line NNRTI for HIV-1-infected patients, according to the current Department of Health and Human Services (DHHS) guidelines [2]. NVP retains its usefulness in antiretroviral-naïve patients due to its lack of teratogenic effects or central nervous system toxicity and a lower cost than efavirenz (EFV) (a preferred initial NNRTI drug). However, one drawback of NVP is its induction of allergic reactions in approximately 5-14% of users worldwide [3-6]. After discontinuing NVP because of this allergic reaction, these patients may begin using other NNRTIs, such as EFV or etravirine (ETR). Most of these patients continue to take EFV even after the allergic reaction to NVP is resolved because of the lower incidence of rash associated with EFV vs. NVP and the requirement of a single daily dose. A recurrent rash has been described in approximately 12.6% (95% confidence interval 2.7–22.4%) of HIV-1-infected patients who previously experienced an allergic reaction to NVP and then began using EFV [7].

The method of antiretroviral treatment interruption following an allergic reaction to an NNRTI-based regimen is a major consideration. The simultaneous discontinuation of all the drugs in a NNRTI-based regimen would result in a period of functional NNRTI monotherapy; NNRTIs exhibit a longer plasma half-life and a longer duration of detectable levels than nucleoside analog reverse transcriptase inhibitors (NRTIs) [8]. NNRTIs exhibit a low genetic barrier to resistance; a single point mutation may lead to high NVP or EFV resistance [9,10]. As of November 3, 2008, the DHHS guidelines [11] recommend that patients either first halt the use of NNRTIs and then continue using NRTIs for a period of time (i.e., staggered interruption) or switch from an NNRTI-based regimen to a protease inhibitor (PI)-based regimen before discontinuing antiretroviral drugs (ARV). The simultaneous interruption of all the drugs in an NNRTI-based regimen is not recommended because it might induce a resistance mutation. This guideline is based primarily on clinical studies on the prevention of mother-to-child transmission [12-14] and treatment interruption strategies [15].

HIV-1-infected patients with a history of allergic reactions to NVP who plan to begin an EFV-based regimen may later experience different effects from those associated with the prevention of mother-to-child transmission or with treatment interruption strategies. Thus, we conducted a retrospective cohort study to examine the effectiveness of different types of treatment interruption strategies in HIV-1-infected patients who halted an NVP-based regimen because of cutaneous allergic reactions to NVP and later began an EFV-based regimen.

## Methods

### Study population

This retrospective cohort study was performed at the Bamrasnaradura Infectious Diseases Institute, Ministry of Public Health, Nonthaburi, Thailand. The Simultaneous Interruption group consisted of HIV-1-infected patients who simultaneously discontinued the use of all the drugs in an NVP-based regimen after experiencing a cutaneous allergic reaction to the treatment. The Staggered Interruption group consisted of HIV-1-infected patients who first discontinued the use of NVP after experiencing a cutaneous allergic reaction to an NVP-based regimen but who continued with other NRTIs for several days. The Control group included patients who began an EFV-based regimen as first-line therapy and who were never exposed to NVP. The study end date was March 2010.

**Figure 1:**
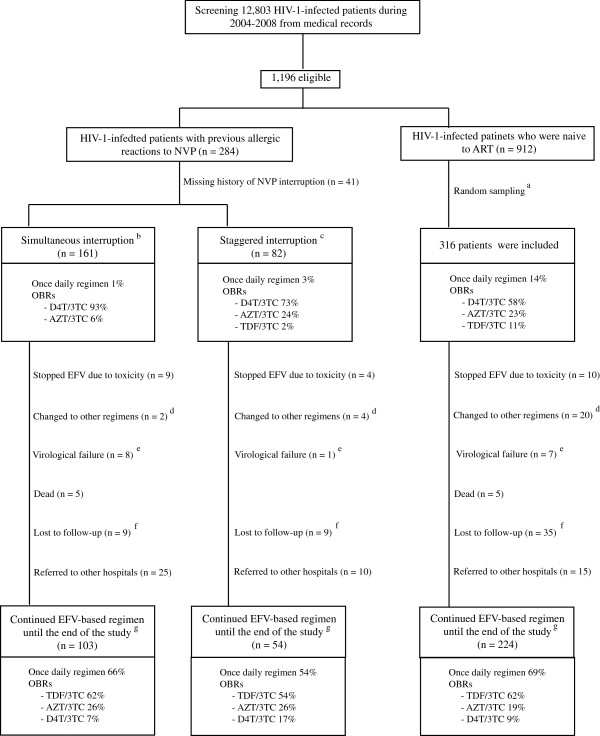
**Profile of the study cohort.** ART = Antiretroviral therapy; AZT = Zidovudine; D4T = Stavudine; EFV = efavirenz; NVP = Nevirapine; OBRs = optimized background regimens; TDF = tenofovir; 3TC = lamivudine. ^a^Random sampling was performed using random numbers. ^b^HIV-1-infected patients who simultaneously discontinued all drugs in NVP-based regimens when they experienced allergic reactions to NVP-based regimens. ^c^HIV-1-infected patients who discontinued NVP first but continued use of the other NRTIs for a few days when they experienced allergic reactions to NVP-based regimens. ^d^This criterion included patients who changed EFV to other NNRTIs or PIs. It did not include patients who only changed or modify NRTIs. ^e^Virological failure was defined as either (1) having two consecutive results of plasma HIV-1 RNA >400 copies/ml after 6 month of a EFV-based regimen or (2) having plasma HIV-1 RNA >1,000 copies/ml plus having any genotypic resistance mutation to efavirenz-based regimen. ^f^It included either (1) patients who did not have clinical visits for more than 3 months from appointment date or (2) patients who stopped efavirenz-based regimen for more than 3 months. ^g^The cohort ended on 31 March 2010.

A total of 12,803 medical records of HIV-1-infected patients were retrieved from a medical database and reviewed (Figure 1). The inclusion criteria were as follows: (a) documented HIV-1 infection, (b) 18–70 years of age, (c) began an EFV-based regimen as the initial treatment or began an EFV-based regimen after the resolution of a cutaneous allergic reaction to the initial NVP-based regimen, and (d) began an EFV-based regimen between January 2002 and December 2008 at the Bamrasnaradura Infectious Diseases Institute. The exclusion criteria were as follows: (a) previously received a non-HAART regimen, such as a dual-NRTI regimen or a single-dose NVP with zidovudine monotherapy, and (b) a history of diseases or conditions that severely affect either kidney or liver function, such as decompensated liver cirrhosis or end-stage renal disease.

The study was approved by the Ethics Committee for Research in Human Subjects, Department of Disease Control, Ministry of Public Health, Thailand.

### Data collection

Data were collected from the medical records of the eligible patients. These data included (a) demographic features, (b) any history of allergic reactions to NVP-based regimens or other drugs, (c) previous and current antiretroviral (ARV) regimens, (d) CD4 cell counts and plasma HIV-1 RNA levels, and (e) both laboratory and clinical adverse events.

Plasma HIV-1 RNA was initially measured using reverse transcriptase polymerase chain reaction (RT-PCR) with a COBAS AMPLICOR HIV-1 MONITOR test, version 1.5 (Roche Molecular Systems, Inc., Branchburg, NJ, USA), which detects plasma HIV-1 RNA levels in the range of 50–100,000 copies/ml (ultrasensitive technique) or 400–1,000,000 copies/ml (standard technique). After June 2008, plasma HIV-1 RNA levels were evaluated using real-time PCR with a COBAS® AmpliPrep/COBAS® TaqMan® HIV-1 test (Roche Molecular Systems, Inc.), which can be used to measure plasma HIV-1 RNA levels in the range of 40–10,000,000 copies/ml.

Patients who did not appear for clinical visits for more than 3 months after a missed appointment or who discontinued an EFV-based regimen for more than 3 months were considered lost to follow-up. Patients who halted an EFV-based regimen for fewer than 3 months were classified as having an EFV-based regimen interruption.

We defined an adverse laboratory event as any laboratory result of at least a grade 3, according to the Division of AIDS Table for Grading the Severity of Adult and Pediatric Adverse Events (the DAIDS AE grading table). A clinical adverse event was defined as any clinical adverse event that resulted in the physician changing or modifying the antiretroviral regimen. A patient was allowed to continue participating in the study if the physician only changed or modified the NRTIs used. However, if the physician changed EFV to other NNRTIs or PIs, the patient was discontinued from the study cohort.

Between January 2010 and March 2010, there was a final evaluation of the HIV-1-infected patients who continued an EFV-based regimen through the end of study. The evaluation consisted of the following: (a) a plasma HIV-1 RNA assay, (b) a CD4 cell count, (c) a complete blood count, and (d) blood chemistry tests. Patients were informed about the evaluation if they (a) came to the hospital during this period, (b) were continuing an EFV-based regimen, and (c) did not have the results of any plasma HIV-1 RNA measurements obtained between October 1, 2009 and December 31, 2009. At the end of the study, 381 patients were still participating (Figure 1). Of these, 205 patients were eligible for the final evaluation because 176 patients had undergone plasma HIV-1 RNA assays between October 2009 and December 2009. There were 201 patients (98%) who completed the evaluation and provided written informed consent for the study. The remaining four patients attended the follow-up examination at the hospital, but we were unable to contact them concerning the evaluation and consent form.

### Statistical analysis

The primary outcome was virological failure, which was defined as either (a) two consecutive plasma assay results indicating HIV-1 RNA levels >400 copies/mL after 6 months of an EFV-based regimen or (b) a single plasma HIV-1 RNA level >1,000 copies/mL in addition to any genotypic resistance mutation to an EFV-based regimen. These criteria included only virological results obtained after 6 months of an EFV-based regimen. Genotypic resistance mutations were included in the virological criteria to reduce the bias from viral blips and noncompliance.

The secondary outcomes were as follows: (a) the percentage of patients who exhibited virological suppression; (b) the CD4 cell counts; (c) the clinical outcomes of death, major opportunistic infection, immune recovery syndrome, and non-AIDS-associated disease; and (d) adverse laboratory and clinical events.

The relative risk and 95% confidence intervals were calculated to compare the differences in primary outcome among the groups. In addition, stratified analysis models were used to evaluate the effects of confounding factors on the primary outcome.

Virological suppression was defined as either (a) a plasma HIV-1 RNA level <50 copies/ml (based on RT-PCR using the COBAS Amplicor HIV-1 Monitor test, version 1.5) or (b) a plasma HIV-1 RNA level <40 copies/ml (based on real-time PCR using the COBAS® AmpliPrep/COBAS® TaqMan® HIV-1 test).

The baseline characteristics and outcomes were analyzed with (a) a chi-squared test for dichotomous data, (b) the Mann–Whitney test or the Kruskal-Wallis test for continuous data with a non-normal distribution, and (c) an unpaired *t*-test and a one-way ANOVA for continuous data with a normal distribution. All the reported *p*-values were two-sided and were considered statistically significant if *p*<0.05. The analyses were performed using SPSS version 14.

## Results

### Participants and baseline demographic features

Figure 1 presents the profile of the study cohort. There were 559 patients at the beginning of the study (January 2002) and 381 patients at the end of the study (March 2010).

Among the 139 patients who (a) were lost to follow-up (n = 53), (b) were referred to other hospitals (n = 50), (c) experienced a change in therapy from EFV to another ARV (n = 26), and (d) died (n = 10), 104 patients (74.8%) underwent plasma HIV-1 RNA assays within 6 months before they were removed from the cohort. Ninety-five of these patients (91.3%) demonstrated virological suppression.

There were 26 patients in the study (4.7%) whose therapy was changed from EFV to another ARV (another NNRTI or PI) (Figure 1). This group did not include patients who (a) received only changes or modifications to their NRTIs or (b) failed an EFV-based regimen or experienced EFV toxicity. The main reasons for substituting another therapy for EFV were financial issues (35%) and pregnancy (23%).

The baseline demographic features of the patients are summarized in Table 1. The patients in the Staggered Interruption group continued using other NRTIs after discontinuing NVP for a median (IQR) duration of 7 (6, 9) days. The NRTIs that were most commonly used in the Staggered Interruption group were stavudine/lamivudine (d4T/3TC, 73.3%) and zidovudine/lamivudine (AZT/3TC, 23.3%).

**Table 1:** **Baseline demographic and clinical characteristics of eligible HIV**-**1-infected patients**

		**EFV-based regimens**			
	**Available data (n)**	**Simultaneous Interruption**^**a**^	**Staggered Interruption**^**b**^	**Controls**^**c**^	***p*value**^**d**^
Total number, n	559	161	82	316	
Mean age, years (SD)	559	41.8 (8.3)	39.4 (9.4)	43.0 (8.4)	0.003
Female, %	559	39.1	59.8	33.9	<0.001
Mean body weight, kilograms (SD)	556	54.9 (9.2)	54.8 (9.8)	56.9 (12.4)	0.091
History of previous major opportunistic infections, %	556	40.6	35.4	51.9	0.007
History of previous allergic reactions to other drugs (exclude NVP), %	555	32.7	30.9	11.4	<0.001
Median duration of previous NVP-based regimens, days (IQR)	234	14 (11,27)	14 (12, 30)		0.574
Median baseline of CD4 cell count, cells/uL (IQR) ^e^	543	69 (24, 140)	90 (26, 167)	47 (13, 132)	0.006
Baseline plasma HIV-1 RNA >100,000 copies/ml, % ^e^	258	69.6	77.4	76.0	0.595
Baseline EFV-based regimens					
-Once-daily regimen,%	556	0.6	2.5	13.7	<0.001
-OBRs	559				
D4T/3TC, %		93	73	58	
AZT/3TC, %		6	24	23	
TDF/3TC, %			2	11	

Note: AZT = Zidovudine; D4T = Stavudine; EFV = Efavirenz; IQR = Interquartile range; NVP = Nevirapine; OBRs = Optimized background regimens; SD = Standard deviation; TDF = Tenofovir; 3TC = Lamivudine.

^a^HIV-1-infected patients who simultaneously discontinued all drugs in NVP-based regimens when they experienced allergic reactions to NVP-based regimens.

^b^HIV-1-infected patients who discontinued NVP first but continued use of the other NRTIs for a few days when they experienced allergic reactions to NVP-based regimens.

^c^HIV-1-infected patients who were naïve to antiretroviral therapy and were never exposed to NVP before beginning EFV-based regimens.

^d^*p*-value represents the difference between the three groups (Simultaneous interruption, Staggered interruption, and Control).

^e^These results would be included if they were examined within 6 months before starting EFV-based regimens.

### Main outcomes

Table 2 summarizes the outcomes of this cohort.

**Table 2:** Results of treatment outcomes with EFV-based regimens in eligible HIV-1-infected patients (n = 559)

		**EFV-based regimens**			
	**Available data (n)**	**Simultaneous Interruption**^**a**^	**Staggered Interruption**^**b**^	**Controls**^**c**^	***p*value**^**d**^
Total number, n	559	161	82	316	
Median duration of EFV-based regimens, months (IQR)	559	57 (27–73)	31 (18–52)	40 (23–65)	<0.001
Once-daily regimen at end of study, %	378	66.0	53.7	69.2	0.097
Documented EFV-based regimen interruption, % ^e^	559	9.3	3.7	2.8	0.007
Frequency of plasma HIV-RNA assays	559				
> 2 times/year, %		14.9	32.9	22.2	0.030
1–2 times/year, %		57.1	48.8	60.4	
< 1 time/year, %		28.0	18.3	17.4	
**Primary outcome**					
Incidence of virological failure, cases per 1,000 person-years	559	12.9	5.4	6.6	
Relative risk of having virological failure when compared to Control group, (95% CI)	559	1.97 (0.62–6.38)	0.83 (0.02–6.43)		
**Secondary outcomes**					
Virological suppression^g^ at 24 (±3) months, % (Per-protocol-analysis)	440	66.9	76.9	68.9	0.411
Median CD4 cell counts at 24 (±3) months, cells/μL (IQR)	327	352 (258–524)	387 (309–458)	340 (237–473)	0.483
Median increase from baseline in CD4 cell counts at 24 (±3) months, cells/μL (IQR)	317	264 (184–374)	292 (220–384)	273 (178–383)	0.647
Major opportunistic infections, %	556	6.83	4.88	5.75	0.812
Paradoxical immune recovery syndrome, %	541	4.55	2.60	2.90	0.603
Malignancy, %	559	1.24	0.00	1.27	0.736
Non-AIDS defining conditions ^h^, %	559	0.62	0.00	0.32	1.000
Death, %	559	3.10	0.00	1.58	0.206

Note: AZT = Zidovudine; CI = Confidence interval; D4T = Stavudine; EFV = Efavirenz; IQR = Interquartile range; NVP = Nevirapine; OBRs = Optimized background regimens; SD = Standard deviation; TDF = Tenofovir; 3TC = Lamivudine.

^a^HIV-1-infected patients who simultaneously discontinued all drugs in NVP-based regimens when they experienced allergic reactions to NVP-based regimens.

^b^HIV-1-infected patients who discontinued NVP first but continued use of the other NRTIs for a few days when they experienced allergic reactions to NVP-based regimens.

^c^HIV-1-infected patients who were naïve to antiretroviral therapy and were never exposed to NVP before beginning EFV-based regimens.

^d^*p*-value represents the difference between the three groups (Simultaneous interruption, Staggered interruption, and Control).

^e^Patients who halted EFV-based regimens for fewer than 3 months were classified as having an EFV-based regimen interruption and remained in the study.

^f^Virological failure was defined as either (1) having two consecutive results of plasma HIV-1 RNA >400 copies/ml after 6 month of a EFV-based regimen or (2) having plasma HIV-1 RNA >1,000 copies/ml plus having any genotypic resistance mutation to EFV-based regimen.

^g^Virological suppression was defined as having plasma HIV-1 RNA either: (1) having plasma HIV-1 RNA <50 copies/ml (based on RT-PCR using the COBAS Amplicor HIV-1 Monitor Test Ver 1.5, Roche Molecular Systems Inc., Branchburg, NJ, USA),or (2) having plasma HIV-1 RNA <40 copies/ml (based on RT-PCR using the COBAS® AmpliPrep/COBAS® TaqMan® HIV-1 test, Roche Molecular Systems Inc., Branchburg, NJ, USA).

^h^Non-AIDS defining conditions included major cardiovascular, renal and hepatic disease outcomes as defined by the Strategies for Management of Antiretroviral Therapy (SMART) Study Group [16].

The overall median (IQR) duration of the follow-up period was 43 (22, 66) months. The Simultaneous Interruption group had a significantly longer follow-up period than the other two groups (*p*<0.001).

There were 27 patients who experienced EFV-based regimen interruptions during the study (Table 2). The Simultaneous Interruption group had more documented regimen interruptions than the other two groups (*p* = 0.007). The median (IQR) duration of the EFV-based regimen interruptions was 42 (12, 60) days. Common causes of the regimen interruptions included (a) financial issues (n =7), (b) toxicity (n = 7), and (c) poor compliance with the medication regimen (n = 7). There were 13 patients who discontinued all ARVs during regimen interruptions, whereas the remaining patients switched to other therapies, such as PI- or NVP-based regimens.

The incidence of virological failure was trended toward being higher in the Simultaneous Interruption group than in the other groups (Table 2), but this difference was not statistically significant. The relative risk of virological failure between the Simultaneous Interruption and Control groups was 1.97 (0.62-6.38), *p* = 0.100. The relative risk between the Simultaneous Interruption and Staggered Interruption groups was 2.38 (0.32–105.75), *p* = 0.224. The Staggered Interruption group displayed virological outcomes comparable to those of the Control group.

There were imbalances in the three groups’ baseline characteristics, such as age, sex, previous history of major opportunistic infections, baseline CD4 cell count, frequency of ARV administration, and history of EFV-based regimen interruptions (Table 1 and Table 2). A stratified analysis was performed to adjust for the effects of these factors on the primary outcome (virological failure). There were no significant differences between the adjusted relative risk ratios and the crude relative risk ratios.

Overall, 12 of the 16 subjects (75%) who experienced virological failure underwent genotypic resistance assays prior to changing to other regimens (Simultaneous Interruption n =6, Staggered Interruption n = 1, and Control n = 5). The mutations detected in these 12 subjects included TAMs (n = 4), M184V mutations (n = 6), and NNRTI-resistance mutations (n = 10). The incidence of NNRTI-resistance mutations in the Simultaneous Interruption group was 8.1 cases/1,000 person-years, which trended toward being higher than the incidences in the Staggered Interruption (5.4 cases/1000 person-years) and Control (3.74 cases/1000 person-years) groups. However, these differences were not statistically significant. The Y181C and G190A NNRTI-resistance mutations, which are frequently related to the selective effect of NVP [17,18], were identified in only one subject in each of the Staggered Interruption and Simultaneous Interruption groups.

At 24 months after EFV-based regimens, there were 440 patients in the cohort. Virological suppression at 24 (±3) months after receiving EFV-based regimens (per-protocol analysis) was not significantly different among the three groups (*p* = 0.411) (Table 2). However, there were only 314 patients (71%) who had virological results during this period, and patients who did not have virological results were counted as non-suppressed when we analyzed for virological suppression.

### Adverse events of EFV-based regimens

Table 3 presents the adverse events associated with EFV-based regimens among the study cohort. There were 23 patients (4.1%) who were unable to tolerate EFV (Figure 1), mainly due to either (a) EFV-induced central nervous system toxicity (n = 10) or (b) EFV-induced allergic reactions (n = 8).

**Table 3:** Adverse events of EFV-based regimens in eligible HIV-1-infected patients (n = 559)

	**EFV-based regimens**			
	**Simultaneous Interruption**^**a**^	**Staggered Interruption**^**b**^	**Controls**^**c**^	***p*value**^**d**^
Total number, n	161	82	316	
***Significant clinical adverse events***^e^				
Lipoatrophy/Lipodystrophy, %	47.2	26.8	31.0	0.001
Mitochondrial toxicity^f^, %	2.5	6.1	0.3	0.001
Severe anemia, %	3.1	2.4	2.9	0.957
Renal toxicity, n (%)	1 (0.6%)	0	0	0.435
CNS toxicity, n (%)	4 (2.5%)	2 (2.4%)	5 (1.6%)	0.6293
Allergic reactions to EFV, n (%)	3 (1.9%)	1 (1.2%)	4 (1.3%)	0.816
Allergic reactions to NRTIs, n (%)	1 (0.6%)	1 (1.2%)	5 (1.6%)	0.672
***≥ Grade 3 laboratory adverse events***^g^				
Diabetes Mellitus^h^, %	3.7	2.4	5.7	0.369
Hypertriglyceride^i^, %	5.6	2.4	2.9	0.261
Hypercholesterolemia^j^, %	8.1	2.4	4.1	0.088
Transaminitis^k^, %	4.4	0.0	1.0	0.027

Note: CNS = Central nervous system; EFV = Efavirenz; NRTI = Nucleoside analogue reverse transcriptase inhibitor.

^a^HIV-1-infected patients who simultaneously discontinued all drugs in NVP-based regimens when they experienced allergic reactions to NVP-based regimens.

^b^HIV-1-infected patients who discontinued NVP first but continued use of the other NRTIs for a few days when they experienced allergic reactions to NVP-based regimens.

^c^HIV-1-infected patients who were naïve to antiretroviral therapy and were never exposed to NVP before beginning EFV-based regimens.

^d^*p*-value represents the difference between the three groups (Simultaneous interruption, Staggered interruption, and Control).

^e^ Significant clinical adverse events was defined as clinical events that caused doctors to change or modify antiretroviral therapy.

^f^Mitochondrial toxicity was diagnosed if a patient had both (1) serum lactate ≥ 2 mmol/L, and (2) symptoms caused by NRTI toxicity such as lipoatrophy, peripheral neuropathy, and lactic acidosis.

^g^These events were diagnosed based on the Division of AIDS Table for Grading the Severity of Adult and Pediatric Adverse Events (“DAIDS AE grading table”).

^h^Diabetes mellitus defined as a new onset with medication initiation is indicated OR uncontrolled DM despite medication modification.

^i^Triglyceride >750 mg/dL.

^j^Cholesterol >300 mg/dL.

^k^either AST or ALT ≥ 185 u/L.

## Discussion

The current guidelines [2,19] recommend the use of a staggered interruption strategy in patients who must interrupt an NNRTI-based regimen. The duration of the use of NRTIs after the discontinuation of the NNRTI regimen ranged from 1 to 3 weeks [2,8,19]. However, the optimal duration has not been determined.

In this study, the patients in the Staggered Interruption group, who continued the use of 2 NRTIs for a median of 7 days after discontinuing NVP, had virological outcomes comparable to those of the Control group (Figure 1 and Table 2). These results are in accordance with a post-study analysis of the Strategies for Management of Antiretroviral Therapy (SMART) trial by Fox et al. [15]. Consequently, it should be appropriate to use a staggered interruption strategy if the NRTIs are to be continued for 7 days.

Among the HIV-infected patients who received an NVP-based regimen for at least 2 weeks, the duration of detectable NVP levels after discontinuation varied from 7 to 21 days [20,21]. Thus, patients who experience acute allergic reactions to an NVP-based regimen may acquire NNRTI-resistant mutations if they simultaneously stop all the drugs in the NVP-based regimen. A simultaneous interruption strategy for discontinuing NNRTI-based regimens is not recommended in the current DHHS guidelines [2]. In this study, there was a higher incidence of virological failure and genotypic resistance mutations to NNRTI in the Simultaneous Interruption group than in the other groups. Thus, a simultaneous interruption strategy is not advisable.

The median follow-up time duration of the Simultaneous Interruption group was significantly longer than those of the other groups (*p*<0.001) (Table 1). This result may have been influenced by a general recommendation in the DHHS guidelines (the November 3, 2008 version) [11] that a staggered interruption strategy should be employed to prevent NNRTI resistance after discontinuing NNRTI-based regimens. In this study, there were 141 (88%) patients in the Simultaneous Interruption group who began an EFV-based regimen prior to November 3, 2008.

The current guidelines recommend that patient plasma HIV-1 RNA levels and CD4 cell counts be monitored regularly, at least twice per year [1,2,22]. Nevertheless, the plasma HIV-1 RNA levels of 21% of the cohort were assayed less than once per year. Thus, the final evaluation was designed to assess the laboratory data of the patients who remained in the cohort through the end of study. The evaluation may have produced a more accurate diagnosis of laboratory-related events and data, such as virological outcomes and laboratory adverse events.

In the beginning of the study, d4T/3TC was commonly administered to our patients (Figure 1) because TDF was not available in our hospital until January 2007.

The low incidence of virological failure may have resulted from the removal from the cohort of patients who were lost to follow-up (e.g., either a documented discontinuation of an EFV-based regimen for more than 3 months or a failure to attend a follow-up examination at the hospital for longer than 3 months after the original appointment date). These patients generally exhibited low compliance with ARV therapy, which is associated with virological failure [23,24].

This study had the following limitations: (a) a retrospective design; (b) a non-randomized design; (c) some incomplete data (including CD4 cell counts, plasma HIV-1 RNA levels, and the results of genotypic resistance assays); (d) a follow-up loss of 9.5%; and (e) the frequent use, in the beginning of the study, of NRTI drugs such as d4T/3TC that are not preferred NRTIs under the current guidelines (1, 2, 22). These limitations should be carefully considered when interpreting the data from this study.

## Conclusion

In our cohort of patients who experienced acute allergic reactions to first-line NVP-based therapy and later began an EFV-based regimen, the virological outcomes of the Staggered Interruption group (who continued 2 NRTIs for 7 days after discontinuing NVP) did not differ from those of the Control group (who began an EFV-based regimen as their initial treatment). However, the virological failure in the Simultaneous Interruption group was possibly higher than that in the Staggered Interruption and Control groups.

## Abbreviations

ABC: Abacavir; ARV: Antiretroviral; AZT: Zidovudine; DHHS: Department of Health and Human Services; D4T: Stavudine; EACS: European AIDS clinical society; EFV: Efavirenz; ETR: Etravirine; NNRTI: Non-nucleoside analogue reverse transcriptase inhibitor; NRTIs: Nucleoside analogue reverse transcriptase inhibitors; NVP: Nevirapine; PI: Protease inhibitor; SMART: Strategies for management of antiretroviral therapy; TDF: Tenofovir; 3TC: Lamivudine.

## Competing interests

Krittaecho Siripassorn received research and/or honoraria/travel grants from Merck, Sharp and Dohme and Bristol-Myers Squibb in the past five years. KiatRuxrungtham received consultancy fees and/or honoraria and travel and/or research grants from Tibotec, F Hoffmann-La Roche, Merck, Sharp and Dohme, Bristol-Myers Squibb, Gilead, Abbott, and Glaxo SmithKline.

## Authors’ contributions

KR and WM prepared drafts of the report and reviewed and edited the final manuscript. AP and SN screened medical records and contributed important information to the methods section. AC, NH, KW, and AL reviewed medical records and provided information to validate the use of the chosen analytical methods. All authors read and approved the final manuscript.
